# Targeting Autophagy to Treat Cancer: Challenges and Opportunities

**DOI:** 10.3389/fphar.2020.590344

**Published:** 2020-11-23

**Authors:** Junghyun Lim, Aditya Murthy

**Affiliations:** Department of Cancer Immunology, Genentech, Inc., South San Francisco, CA, United States

**Keywords:** autoph, agy, immunotherapy, cancer, oncology, immunology

## Abstract

Autophagy is a catabolic process that targets its cargo for lysosomal degradation. In addition to its function in maintaining tissue homeostasis, autophagy is recognized to play a context-dependent role in cancer. Autophagy may inhibit tumor initiation under specific contexts; however, a growing body of evidence supports a pro-tumorigenic role of this pathway in established disease. In this setting, autophagy drives treatment resistance, metabolic changes, and immunosuppression both in a tumor-intrinsic and extrinsic manner. This observation has prompted renewed interest in targeting autophagy for cancer therapy. Novel genetic models have proven especially insightful, revealing unique and overlapping roles of individual autophagy-related genes in tumor progression. Despite identification of pharmacologically actionable nodes in the pathway, fundamental challenges still exist for successful therapeutic inhibition of autophagy. Here we summarize the current understanding of autophagy as a driver of resistance against targeted and immuno-therapies and highlight knowledge gaps that, if addressed, may provide meaningful advances in the treatment of cancer.

## Introduction

Autophagy is an evolutionarily conserved lysosomal degradative pathway that digests diverse cellular cargo. These include cytosol itself, proteins, lipids, organelles and intracellular pathogens. Given its role in cellular quality control, autophagy is unsurprisingly involved in numerous pathophysiological conditions. Since Christian de Duve coined the term “autophagy,” our understanding of this pathway has evolved from a relatively simple non-selective catabolic process to a highly targeted mechanism by which specific cargo is identified for lysosomal turnover (Klionsky et al., 2003; Dikic and Elazar, 2018; Mizushima, 2018; Levine and Kroemer, 2019; Melia et al., 2020). Selective autophagy utilizes “autophagy receptor” proteins which bridge the cargo-of-interest to the autophagosome (reviewed in Kirkin, 2020). Each form of selective autophagy is named after its cargo (e.g., protein aggregates: aggrephagy, lipids: lipophagy, pathogens: xenophagy, organelles: mitophagy, pexophagy, ribophagy, ER-phagy, and nucleophagy). Autophagy receptors such as p62/SQSTM1, NDP52, OPTN and NBR1 consist of a ubiquitin-associated-domain and an LC3-interacting region. This allows them to bridge specific cargo (often ubiquitinated as a consequence of a specific cellular state/stress response) to autophagosomes by binding to members of the ATG8 family, such as LC3 (Zaffagnini and Martens, 2016; Kirkin and Rogov, 2019). Three types of autophagy have been characterized based on how cargo is delivered to the lysosome: macroautophagy, microautophagy, and chaperone-mediated autophagy (CMA). Macroautophagy is the most extensively studied form of autophagy and the focus of this review. Molecular mechanisms underlying microautophagy and CMA are reviewed in detail elsewhere (Kaushik and Cuervo, 2018; Oku and Sakai, 2018).

**FIGURE 1:**
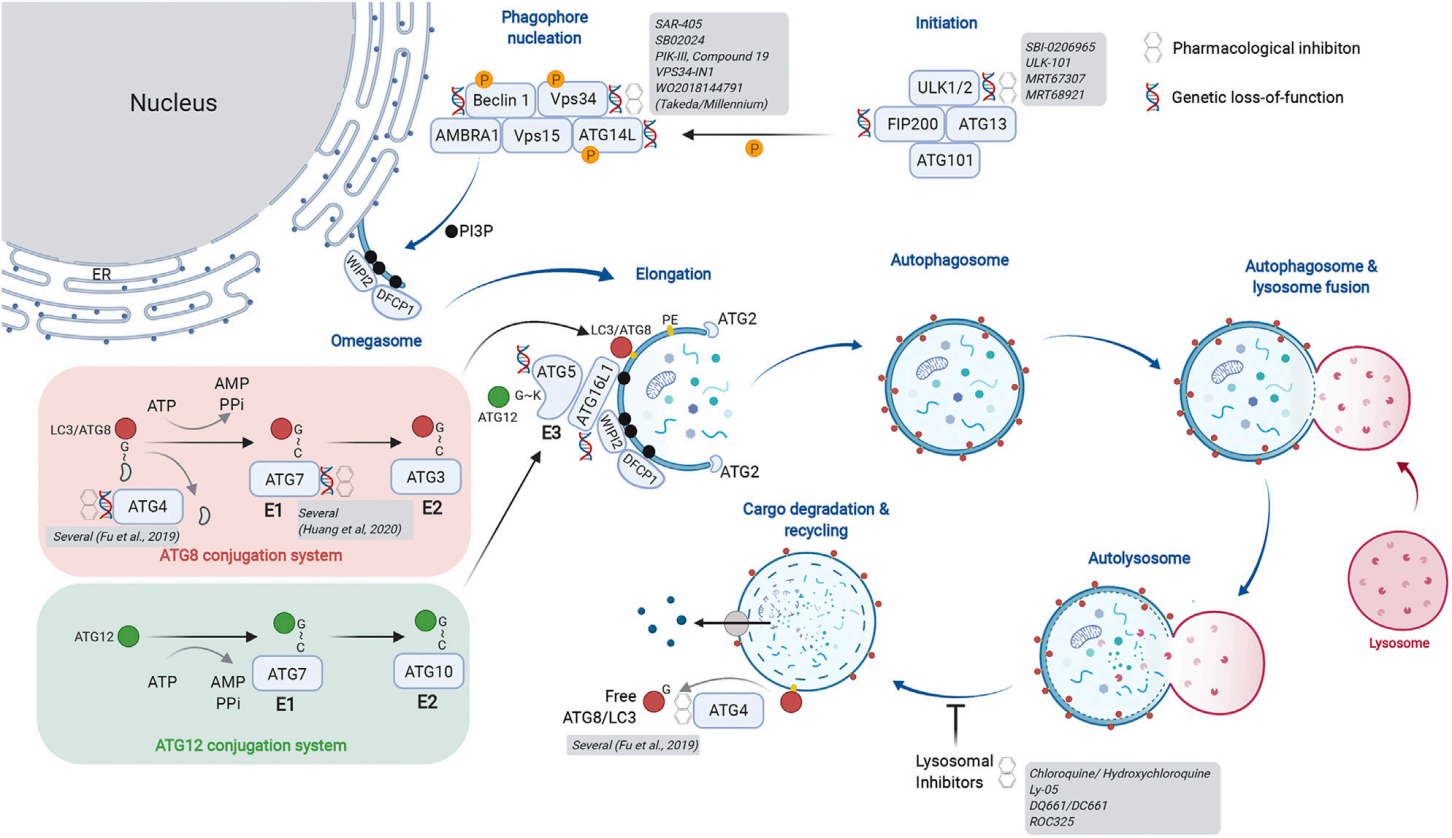
Actionable nodes of the autophagy pathway. The process of autophagy begins with generation of the initiation complex. Its substrates include factors critical for phagophore nucleation. This is also called the Vps34 complex and is involved in initiation of autophagy as well as endosome maturation. The lipid kinase activity of Vps34 generates PI3P on the target membrane, forming the omegasome upon which the autophagy nucleation complex forms. The ER is depicted as a membrane source for the omegasome, but other membranous compartments have also been described. The autophagy elongation machinery includes an E1-E2-E3-like process that ultimately results in lipidation of ATG8 family proteins, thereby identifying a mature autophagosome. Two conjugation systems are illustrated: the ATG8 conjugation system (red) transfers LC3/ATG8 to the E2-like protein ATG3. The ATG12 conjugation system (green) generates the E3-like complex by transferring ATG12 onto ATG5. Association of the ATG5-12 fusion with ATG16L1 forms the ATG16L1 complex; this acts in an E3-like manner to transfer ATG8/LC3 onto phosphatidyl ethanolamine (PE), thereby completing the lipidation process. Cysteine proteases of the ATG4 family cleave ATG8/LC3 from the cytosolic face of the autolysosome for re-use. ATG4 family proteins are critical in the initial activation of ATG8/LC3 by exposing the C-terminal glycine for conjugation. As depicted, several members of core autophagy machinery have been assessed in genetic and pharmacological models for their role in cancer biology. Additional details of genetic models are provided in Table 1. A more extensive list of pharmacological inhibitors is provided in Table 2. Created with BioRender.com.

Macroautophagy (herein autophagy) is regulated in a stepwise manner by multi-subunit complexes of autophagy-related proteins (Figure 1). The Unc51-like kinase (ULK) complex is a Ser/Thr protein kinase complex that phosphorylates multiple substrate proteins to initiate autophagy. ULK1/2 activity promotes stabilization of the Class lll phosphatidylinositol 3-kinase (PIK3C3) complex, where Vps34 phosphorylates phosphatidylinositol (PI) to generate PI-(3)-phosphate (Ptdins(3)P), a critical membrane targeting signal for the autophagosome elongation machinery. Importantly, Vps34 forms different subcomplexes with distinct functions. Complex l consists of Beclin 1, p150, ATG14L, and NRBF2 and regulates autophagy induction. Separately, complex II utilizes UVRAG instead of ATG14L and governs vesicular trafficking. Finally, complex III consists of RUBICON, a RUN-domain containing protein that binds UVRAG and negatively regulates autophagosome formation via regulation of the RAB7-GTPase (Matsunaga et al., 2009; Zhong et al., 2009; Ohashi et al., 2019; Bhargava et al., 2020). Thus, Vps34 functions as an upstream regulator of autophagosome formation, endosome maturation and membrane trafficking via its membership in distinct signaling complexes.

Membrane PI3P generated by Vps34 are docking sites for FYVE domain containing proteins such as WIPI2, which in turn recruits the autophagosome elongation complex. Here, ubiquitin-like proteins of the ATG8 family (ATG8 in yeast; LC3A, B, C, GABARAPs, and GATE-16 proteins in mammals) are post-translationally modified by lipidation, first by exposure of a C-terminal glycine and subsequent conjugation to phosphatidylethanolamine (PE). There are parallels between the ubiquitin E1-E2-E3 conjugation machinery and the autophagosome elongation complex. ATG7 acts as the “E1” enzyme for its substrates ATG8 or ATG12. Following E1-mediated activation, ATG8 is transferred to the “E2-like” protein ATG3. The ATG12-ATG5-ATG16L1 complex acts as an “E3-like” enzyme to transfer ATG8 to PE on the growing autophagosome membrane. Importantly, generation of the ATG12-ATG5-ATG16L1 complex requires transfer of ATG12 from ATG7 using a separate conjugation machinery involving ATG10, another “E2-like” protein. Once autophagosomes enclose cargo, they undergo heterotypic fusion with lysosomes for substrate degradation by lysosomal hydrolases. Finally, ATG4 family proteins act as cysteine proteases to cleave and expose C-terminal glycine on ATG8 proteins (e.g., G120 of pro-LC3B) for participation in the elongation complex. They also cleave lipidated ATG8 proteins on the mature autophagosome to recycle them and maintain a cellular pool of non-lipidated ATG8. As depicted in Figure 1, our current understanding of the autophagic machinery provides multiple nodes that are genetically validated and attractive pharmacological targets.

This review summarizes our current understanding of how autophagy drives disease progression via altered metabolism and immunosuppression in tumor-intrinsic and extrinsic contexts. We catalogue the growing list of actionable targets in the pathway, and finally discuss current gaps in knowledge to successfully modulate autophagy for the treatment of cancer.

## The Complicated Role of Autophagy in Cancer

Initial genetic studies using *Becn1* (encoding Beclin 1) heterozygous mice demonstrated spontaneous malignancies and HBV-induced hepatocellular carcinogenesis in murine models (Qu et al., 2003; Yue et al., 2003). Autophagy activity in *Becn1* ^+/−^ mice was reduced compared to controls. Since outgrowing tumors did not show loss of the remaining allele, *Becn1* was proposed as a haplo-insufficient tumor suppressor. Comparatively, deletion of downstream autophagy genes *Atg5* or *Atg7* permitted development of benign tumors that failed to progress to malignant cancers (Takamura et al., 2011; Guo et al., 2013; Strohecker et al., 2013; Rao et al., 2014; Yang et al., 2014). Loss of the autophagy-related gene *Atg7* in intestinal epithelial cells was shown to attenuate tumor growth driven by loss of adenomatous polyposis coli (APC), a major tumor suppressor in colorectal cancer. However, simultaneous deletion of *Atg7* and the tumor suppressor *Tp53* initiated adenoma formation in the presence of wild type APC (Levy et al., 2015; Trentesaux et al., 2020). Thus, loss of autophagy-related genes can promote opposing outcomes for tumor growth depending on the driving oncogenic or tumor-suppressor. Defective autophagy can promote tumor initiation via multiple pathways. For instance, tumor cells derived from autophagy-deficient cells frequently exhibit accumulation of abnormal mitochondria due to lack of mitophagy, consequently suffering enhanced oxidative stress, DNA damage and potentially developing aneuploidy (Mathew et al., 2009). Autophagy also impacts cellular senescence in a context dependent manner. As a key feature of aging, senescence prevents malignant transformation by arresting cell division. While basal autophagy may counteract senescence by maintaining cellular fitness, autophagy is associated with oncogene-induced senescence (Dou et al., 2015; Kang et al., 2015). Autophagy-mediated turnover of p62/SQSTM1 (p62) can also contribute to tumor suppression. As an autophagy receptor, p62 bridges cargo to autophagosomes, but itself is a substrate for autophagic turnover. A known non-canonical function of p62 is regulation of the transcription factor Nrf2 (nuclear factor erythroid 2–related factor 2; discussed later) (Komatsu et al., 2010; Inami et al., 2011; Ichimura et al., 2013). A relatively limited number of studies suggest autophagy as a tumor suppressor in a cancer cell-extrinsic manner. p62 in the tumor microenvironment (TME), specifically in CAFs or adipocytes appears to suppress tumor growth (Valencia et al., 2014; Duran et al., 2016; Umemura et al., 2016). Autophagy has also shown to promote dendritic cell-mediated CD8^+^ T cell priming, thus enhancing tumor cell killing through immunogenic ATP release (Michaud et al., 2011).

In contrast to its role as a tumor suppressor, an increasing number of studies propose a pro-tumorigenic role for autophagy in a tumor cell-intrinsic or extrinsic manner. While some tumor cells induce autophagy as a survival mechanism against diverse stress conditions including therapies, nutrient deprivation, or hypoxia, pancreatic cancer cells exhibit elevated autophagy activity even under basal conditions (Guo et al., 2011; Lock et al., 2011; Yang et al., 2011). Initial observations that autophagy was dispensable for basal growth of multiple cancer cell lines in 2D cell culture systems or xenograft models led to a pause in the pursuit of autophagy inhibitors (Eng et al., 2016). However, emerging data using more relevant model systems are proving that enhanced autophagy is indeed a critical driver of treatment resistance and tumor progression *in vivo* (Table 1)*.* The use of nutrient-rich 2D culture systems and immunocompromised models may account for discrepancies observed between earlier studies and more recent work. Earlier studies of germline autophagy-related gene knockout mice showed autophagy to be a vital pathway for mammalian development, maintenance and expansion of immune cells and hematopoietic stem cells (reviewed in Kuma et al., 2017; Clarke and Simon, 2019); it was thus assumed that autophagy inhibition would compromise immune cell function in the context of cancer. However, recent work using elegant genetic models reveals that in fact, autophagy suppresses anti-tumor immunity. As listed in Table 1, tissue-specific deletion of autophagy-related genes in the myeloid, lymphoid and broader host cellular compartments have demonstrated that perturbing the pathway accelerates tumor clearance via multiple mechanisms (Table 1A – tumor intrinsic; Table 1B – tumor extrinsic). The following sections discuss our emerging understanding of how autophagy promotes therapeutic resistance and fosters tumor progression.

**TABLE 1:** Murine models where loss-of-function in autophagy genes attenuates tumor growth.

A) Autophagy deficiency in the tumor						
Autophagy function	Autophagy- related gene	Cell line/**GEMM**	Cancer type	Tumor Progression	Related mechanism	References
Autophagy initiation	*Fip200*	**MMTV-PyMT**	Breast	Attenuated (extended survival)	Lung metastasis ↓; IFNγ + CD8 ↑; Treg ↓; CCL5, CXCL9/10 ↑	(Wei et al., 2011)
Phagophore nucleation	*Pik3c3* (Vps34)	B16-F10, CT26	Melanoma, CRC	Attenuated (extended survival)	—	(Noman et al., 2020)
	*Becn 1* (Beclin 1)	B16-F10	Melanoma	Attenuated	CCL5-mediated infiltration of NK cells	(Mgrditchian et al., 2017)
Autophagosome elongation	*Atg4B* ^*C47A*^	Mia-Paca2	PDAC	No effect	Tumor regression in MEKi combo	(Kinsey et al., 2019)
	*Atg4B* ^*C47A*^ *, Atg7*	HY15549, HY19636	PDAC, liver metastasis	Attenuated	MHC-l ↑; CD8^+^ T-cell mediated synergy with αPD1 + αCTLA4	(Yamamoto et al., 2020)
	*Atg4B* ^*C47A*^	***LSL-Kras*** ^***G12D***^ **; *Trp53*** ^***lox/+***^ ***; p48Cre*** ^***+***^	PDAC	Regressed (Extended survival)	Comparable tumor control to intermittent expression;Increased Macs	(Yang et al., 2014)
	*Atg5*	8988T	PDAC	Attenuated	—	(Yang et al., 2011)
	*Atg5*	***LSL-Kras*** ^***G12D***^	Lung	Attenuated	Tumor Initiation ↑; Extended survival	(Rao et al., 2014)
	*Atg5*	***LSL-Kras*** ^***G12D***^ **; *Trp53*** ^***lox/+***^ ***; Pdx1Cre*** ^***+***^	PDAC	Attenuated	Initiation ↑;OCR ↓	(Yang et al., 2014)
	*Atg7*	***Braf*** ^***V600E***^ ***; Trp53*** ^***f/f***^	Lung	Attenuated	Initiation ↑; OCR ↓	(Strohecker et al., 2013)
	*Atg7*	***Tg*** ^***Tyr-cre/ERT2/+***^ ***; Lsl-Braf*** ^***V600E/+***^ ***; Pten*** ^***f/+***^	Melanoma	Attenuated	—	(Xie et al., 2015)
	*Atg7*	***VilCre-ER*** ^***T2***^ ***; Apc*** ^***f/+***^	Colon	Attenuated	IFNγ+CD8 ↑; AMPK/p53−mediated	(Levy et al., 2015)
	*Atg7*	***LSL-Kras*** ^***G12D***^ ***, Trp53*** ^***f/f***^	Lung	Attenuated (Extended Survival)	FAO ↓;Glycolysis ↑	(Guo et al., 2013)
	*Multiple*	*Renca, EMT6*	Renal, breast	Attenuated	Sensitization to CTL-mediated killing	(Lawson et al., 2020)

B) Autophagy deficiency in the host							
Autophagy- related gene	Cell line/**GEMM**	Cancer type	Tumor Progression	Related mechanism	References	
*Becn 1, Atg7, Atg5, Rubcn*	B16-F10 (Subcu/IV), LLC	Melanoma/Lung Metastasis,Lung	Attenuated	Myeloid cell specific deletion;STING-mediated Type I IFNs;Enhanced CTL function		(Cunha et al., 2018)
*Atg5, Atg16L1, Atg14L*	E0771, Tramp-C2, MC38	Breast, Prostate, colon	Attenuated	Inducible systemic deletion;IFNγ + TNFα + CD8 T ↑; Glucose uptake ↑		(DeVorkin et al., 2019)
*Atg7*	**frtKras** ^**G12D**^; **Trp53** ^**frt/frt**^	Lung	Attenuated	Inducible systemic deletion;Attenuated growth of established tumor		(Karsli‐Uzunbas et al., 2014)
*Atg7* or *Atg5*	YUMM1.1/1.3, MB49, 71.8	Melanoma, Bladder, NSCLC	Attenuated	Inducible systemic deletion;Serum Arginine depletion by increased ARG1 from liver;Liver-specific deletion phenocopies systemic deletion		(Poillet-Perez et al., 2018)
*Atg7*	MC38	Colon	Attenuated	Deletion in Treg; mTOR, c-Myc, and glycolysis-mediated Treg dysfunction		(Wei et al., 2016)
*Atg7, Fip200*	MB49, YUMM1.1-9	Urothelial carcinoma, Melanoma	Attenuated	Inducible systemic deletion;Enhanced CTL- and STING-mediated inflammatory responses		(Laura Poillet-Perez et al., 2020)

This table provides a summary of genetic models where core autophagy genes have either been deleted or altered to generate loss-of-function/dominant negative phenotypes in autophagic flux. **A)** Earlier focus on the role of autophagy in cancer cells has generated a diverse array of tumor models where autophagy loss-of-function is generated either in transformed murine or human cell lines (underlined) or in genetically engineered mouse models of spontaneous tumor growth (bold) **B)** More recently, loss of autophagy in host cells has been studied using tissue-specific or inducible genetic models. These include gene deletion in the myeloid and lymphoid compartments, as well as broad deletion in multiple tissues via administration of tamoxifen in adult mice. GEMM, Genetically Engineered Mouse Model; Macs, Macrophages; OCR, Oxygen Consumption Rate; FAO, Fatty Acid Oxidation; CTL, Cytotoxic T Lymphocyte.

**TABLE 2:** Pharmacological inhibitors of autophagy.

Autophagy function	Target	Inhibitor name	References
Autophagy initiation	ULK1/2	SBI-0206965	(Egan et al., 2015)
		MRT67307, MRT68921	(Petherick et al., 2015)
		ULK-101	(Martin K. R. et al., 2018)
Phagophore nucleation	Vps34	SAR405	(Ronan et al., 2014)
		PIK-III, compound 19	(Dowdle et al., 2014)
		VPS34-IN1	(Bago et al., 2015)
		SB02024	(Noman et al., 2020)
		Takeda/Millennium	WO2018144791
Autophagosome elongation	GRAMD1A	Autogramin	(Laraia et al., 2019)
	ATG7	Several	(Huang et al., 2020)
	ATG4	NSC185058	(Akin et al., 2014)
		Tioconazole	(Liu et al., 2018)
		UAMC-2526	(Kurdi et al., 2017)
		S130	(Fu et al., 2019)
Cargo degradation	Lysosome	CQ/HCQ	(Levy et al., 2017, Amaravadi et al., 2019)
		Ly05	(McAfee et al., 2012)
		DQ661/DC661	(Rebecca et al., 2017; Rebecca et al., 2019)
		ROC-325	(Nawrocki et al., 2019)

A non-exhaustive list of small molecule inhibitors currently being assessed for autophagy inhibition. The large majority of these are in pre-clinical stages, with the exception of CQ/HCQ. In addition to modulators of known core autophagy-related genes, autophagy pathway inhibitors that target new substrates (e.g., GRAMD1A) are being revealed via phenotypic screening in cells.

## Tumor-Intrinsic Autophagy

### Autophagy Supports Tumor Cell Metabolism

Autophagy supplies tumor cells with metabolic substrates by degrading glycogen, lipid droplets, damaged proteins or organelles (Guo et al., 2011; Yang et al., 2011; Guo et al., 2013; Strohecker et al., 2013; Rao et al., 2014; Yang et al., 2014). In addition, autophagy maintains the pool of functional mitochondria, critical for survival under nutrient or oxygen limiting conditions, and especially for fatty acid oxidation. Accumulation of lipid droplets and defective mitochondria by *Atg7* deficiency attenuated Ras-driven tumor proliferation (Guo et al., 2013; Strohecker et al., 2013). *Atg7* loss also attenuated Braf^V600E^-driven lung tumorigenesis by depleting glutamine, thereby impairing mitochondrial respiration. Inhibition of autophagy by knocking down *Atg7* and *Atg12* attenuated cancer cell glycolysis and cellular transformation by oncogenic *Ras* (Lock et al., 2011). Reduced glucose uptake was also observed upon *Fip200* deletion in the MMTV-PyMT breast cancer model where tumorigenesis is driven by Polyoma virus-mediated Ras, Src, and PI3K activation (Wei et al., 2011). Inhibition of autophagy in tumor cells could therefore sensitize them to metabolic stresses and uncover additional dependencies for survival.

### Autophagy Promotes Therapeutic Resistance

Responses to primary therapy include ER stress, hypoxia, and mTOR inhibition, all of which induce autophagy as a survival mechanism (Rebecca and Amaravadi, 2016; Amaravadi et al., 2019). Hence, autophagy inhibition may reverse resistance to targeted therapies but be comparatively less efficacious on its own. Specific driver mutations such as *Ras, BRAF, LKB1* are known to sensitize cancer cells to autophagy inhibition (Amaravadi et al., 2019). In particular, *Ras*-driven pancreatic cancer is a relevant indication where enhanced autophagy is observed upon pharmacological MAP kinase pathway inhibition (Bryant et al., 2019; Kinsey et al., 2019; Lee et al., 2019). Similarly, *BRAF*-mutations also promote autophagy upon treatment with BRAF or MEK inhibitors (Levy et al., 2014; Ma et al., 2014; Mulcahy Levy et al., 2014; Xie et al., 2015; Mulcahy Levy et al., 2017). In both these settings, combining inhibitors of growth factor signaling along with autophagy provides a synergistic effect, revealing therapeutic efficacy in otherwise non-responsive disease. More recently, selective autophagy of MHC-I by pancreatic cancer cells was shown to promote resistance to immunotherapy, with genetic or pharmacological inhibition of autophagy re-sensitizing cancer cells to checkpoint blockade (Yamamoto et al., 2020). However, class I MHC as well as co-stimulatory or co-inhibitory proteins (e.g., CD40, CD80/86, PD-L1/2) are established interferon response genes. Autophagy is acknowledged as a potent inhibitor of type I and II interferon responses (Martin P. K. et al., 2018; Samie et al., 2018; Orvedahl et al., 2019; Wang et al., 2019; Wang Y. T. et al., 2020; Lawson et al., 2020). Thus, numerous autophagy-dependent mechanisms may govern cellular MHC-I levels. It will be important to confirm whether this observation is unique to pancreatic tumors or a broader phenomenon, given that loss of antigen presentation by tumor cells is a highly relevant mechanism for resistance to immunotherapy (Zaretsky et al., 2016; Patel et al., 2017; Burr et al., 2019).

### Autophagy Protects Against Cell Death

Autophagy can provide tumor cells protection against cell death (Guo et al., 2011; Lock et al., 2011). Immunogenic death of tumor cells by pyroptosis (Wang Q. et al., 2020; Zhang et al., 2020) and necroptosis (Yatim et al., 2015; Snyder et al., 2019) is understood to enhance cellular immunity against cancer by multiple mechanisms including antigen release and adjuvant effects generated by dying cells. Intriguingly, a recent genome-wide assessment of cancer cell-intrinsic mechanisms of resistance against T cell mediated killing revealed autophagy to be a common cyto-protective pathway in murine models (Lawson et al., 2020). Autophagy inhibits necroptosis in intestinal epithelial and innate immune cells by regulating turnover of key components such as RIPK1 and RIPK3 (Matsuzawa-Ishimoto et al., 2017; Lim et al., 2019). In contrast, autophagosomes are shown to provide a platform for the necrosome in prostate epithelial and rhabdomyosarcoma cells (Basit et al., 2013; Goodall et al., 2016). The extent to which this occurs in additional cell types and its significance *in vivo* remains to be determined. Overall, there is consensus that autophagy is a relevant mechanism for cancer cell evasion of cell death (reviewed in Towers et al., 2020), but we have limited understanding of the clinical setting(s) where this dependency can be safely exploited.

### Autophagy Impacts Immune Cell Infiltration of Tumors

Autophagy was reported to coordinate IL-6 secretion in RAS-driven invasion (Lock et al., 2014). Also, non-classical protein secretion was proposed as a mechanism connecting autophagy activity with increased secretion of cytokines known to modulate inflammation and tumorigenesis (Kraya et al., 2015). However, deletion of core autophagy-related genes in immune and cancer cells consistently enhances release of pro-inflammatory cytokines and chemokines (Saitoh et al., 2008; Wei et al., 2011; Murthy et al., 2014; Maycotte et al., 2015; Lee et al., 2016; Mgrditchian et al., 2017; Samie et al., 2018; DeVorkin et al., 2019; Lim et al., 2019; Cotzomi-Ortega et al., 2020; Noman et al., 2020). For example, Mgrditchian et al demonstrated that CCL5 was critical for NK cell infiltration and efficacy in B16F10 melanoma tumors. This is highly relevant for immunotherapy, since the above chemokines are critical for T and NK cell infiltration into solid tumors and strongly associated with a response to immune checkpoint inhibition (Dangaj et al., 2019; House et al., 2020). Thus, inhibition of tumor cell autophagy can reshape the TME by enhanced immune cell recruitment and function.

## Tumor-Extrinsic Autophagy

### Host Autophagy Feeds Tumors

The TME governs several aspects of disease progression (Sahai et al., 2020). Autophagy in the local TME as well as distal host tissues can promote tumor growth by providing critical nutrients. Studies in diverse model systems have shown that autophagy in the host TME epithelial cells (*D. melanogaster* tumor progression models), stromal cells (murine stellate cells, pancreatic ductal adenocarcinoma orthotopic grafts), or distal tissue (murine hepatocytes, subcutaneous melanoma grafts) provides amino acids such as alanine and arginine to support tumor cell survival (Karsli-Uzunbas et al., 2014; Sousa et al., 2016; Katheder et al., 2017; Poillet-Perez et al., 2018). These studies indicate that established tumors enhance autophagic flux in the host, thus shifting the metabolic set-point of numerous cell types including the immune system. This metabolic competition often favors the tumor, since co-opting host amino acids and glucose directly supports tumor growth while crippling the effector function of potentially tumoricidal lymphocytes such as cytotoxic T cell and NK cells (Chang et al., 2015; Mah et al., 2017; Konjar and Veldhoen, 2019; Terren et al., 2019). It is important to note that not all models exhibit sensitivity to autophagy inhibition in the host. Similarly, modulation of adaptive immunity may not play a role in autophagy-mediated tumor growth in certain settings (Poillet-Perez et al., 2018). Thus, it is critical to assess the impact of a specific autophagy-related gene across multiple models to determine its impact on tumor progression, metabolic reprogramming and immunosuppression.

### Autophagy Reprograms Innate Immune Cells

Myeloid cells include macrophages, dendritic cells, monocytes and granulocytes and comprise the innate immune response. As professional phagocytes and antigen presenting cells, macrophages and dendritic cells are critical for shaping the tumor cytokine milieu as well as antigen-specific immunity. Myeloid cell-specific deletion of genes involved in autophagosome elongation and LC3-associated phagocytosis [LAP, reviewed in (Heckmann and Green, 2019)] enhanced anti-tumor immunity, attenuating tumor growth and metastasis in multiple syngeneic tumor models (Alissafi et al., 2018; Cunha et al., 2018). Here, defective autophagy was primarily generated in macrophages, monocytes and granulocytes, resulting in a preferential inflammatory or “M1” polarization of macrophages along with reduced suppressive capacity of myeloid derived suppressor cells. The impact of these changes included enhanced type I interferon responses along with an increase in polyfunctional cytotoxic T lymphocytes. Similar outcomes were observed upon pharmacological inhibition of lysosomal activity using Chloroquine [CQ; (Chen et al., 2018)]. In contrast, loss of Vps34 (encoded by *Pik3c3*) in dendritic cells as well as monocytes/macrophages promoted lung metastasis by B16F10 melanoma cells (Parekh et al., 2017). The broad regulatory role of Vps34 in membrane trafficking beyond autophagy may explain the divergent phenotypes generated by loss of *Pik3c3* versus downstream autophagy-specific genes such as *Atg5*. These findings also reinforce the importance of comparing multiple autophagy pathway genes within a model system to determine if altered autophagy truly underlies the phenotypic outcome. Beyond anti-tumor immunity, deletion of autophagy-related genes in myeloid cells has resulted in enhanced type I/II interferon response, autoimmunity and anti-microbial immunity in numerous studies (Marchiando et al., 2013; Martin P. K. et al., 2018; Samie et al., 2018; Wang Y. T. et al., 2020). Altogether, accumulating evidence strongly supports inhibition of autophagy and related pathways as a mechanism to promote innate inflammation by myeloid cells.

### Autophagy Regulates T Cell Function

Constitutive deletion of core autophagy-related genes in the T lymphocyte lineage has demonstrated its requirement for the development of thymocytes and peripheral T cells, along with maintenance of regulatory T cells (Tregs) (Pua et al., 2007; Kabat et al., 2016; Parekh et al., 2017). Thymocytes rapidly induce autophagy upon TCR engagement to meet bioenergetic needs (Stephenson et al., 2009; Hubbard et al., 2010). Additionally, loss of autophagy in T cells has shown to compromise their ability to generate antigen-specific memory following infection (Puleston et al., 2014; Xu et al., 2014). Intriguingly, defective autophagy in Tregs or in adult mice dramatically enhances anti-tumor immunity via loss of Tregs or generation of a potent effector memory T cell pool in pre-clinical models (Xu et al., 2014; DeVorkin et al., 2019). Recent studies have highlighted the need for sophisticated genetic models to delineate the role of autophagy in lymphocyte developmental versus function in adult tissues (DeVorkin et al., 2019; Laura Poillet-Perez, 2020). DeVorkin et al demonstrated that inducible deletion of *Atg5* in adult mice enhanced T cell glycolytic metabolism while maintaining oxidative phosphorylation (OXPHOS). This shifted CD8^+^ T cells to an effector memory phenotype with increased IFNγ and TNFα production, consistent with the known requirement of glycolysis for optimal effector T cell function (Cham et al., 2008; Chang et al., 2013; Gubser et al., 2013). Notably, inhibition of T cell checkpoints such as PD-1 (encoded by *Pdcd1*) also promotes a shift toward glycolytic metabolism, and germline loss of *Pdcd1* or its ligand PD-L1 impairs T cell memory while driving a terminally differentiated effector phenotype. In contrast, pharmacological PD-1 inhibition drives effector T lymphocyte functions while augmenting memory as the pharmacodynamic effect of PD-1 inhibition wanes (Odorizzi et al., 2015; Patsoukis et al., 2015; Ahn et al., 2018; Verma et al., 2019; Pauken et al., 2020). It appears that enhanced autophagy in the T cell compartment during tumor progression generates a competitive disadvantage for limiting metabolites, thereby curbing productive anti-tumor effector function. It must also be acknowledged that regulation of glycolytic metabolism promotes the maintenance of memory T cell pools (Sukumar et al., 2013; Zhang and Romero, 2018). Thus, timing and duration of autophagy inhibition should be key considerations to optimally induce an effector T cell response against cancer while retaining the ability to develop T cell memory against relevant tumor antigens.

## Adaptation Against Autophagy Inhibition

While tumor cells can induce autophagy as a survival mechanism against therapies, compensatory responses to inhibition of autophagy are also being revealed. For instance, a reversible model of *Atg5* deletion showed that while *Atg5* deficient mice (*ATG5i*) exhibited tissue inflammation and degeneration, eventually succumbing to these phenotypes, there was no sign of overt tumor development (Cassidy et al., 2020). These mice also showed accelerated aging that was reverted by restoring *Atg5* expression (R-Atg5i). Of note, restoring autophagy promoted spontaneous tumor development, suggesting that prolonged autophagy inhibition may select for additional stress-response pathways which accelerate disease progression when autophagy is restored. Recent studies demonstrate that overactivation of the oxidative stress response by the transcription factor *Nrf2* is a dominant consequence of autophagy-related gene deletion in cancer cells (Kerins et al., 2019; Towers et al., 2019). The association between autophagy inhibition and *Nrf2* was reported even earlier, predominantly due to accumulation of p62 (Komatsu et al., 2010; Inami et al., 2011; Ichimura et al., 2013). The Nrf2-Keap1 (kelch-like ECH-associated protein 1) pathway is a critical defense mechanism against oxidative stress. Keap1 tightly regulates Nrf2 activity by promoting its proteasomal degradation under basal conditions. Upon oxidative stress, post-translational cysteine modification of Keap1 unleashes Nrf2, where it induces an anti-oxidant transcriptional program. p62 binds Keap1 at the Nrf2-binding site, and over-abundant p62 outcompetes Nrf2, leading to non-canonical activation of Nrf2-mediated transcription (Komatsu et al., 2010). Nrf2-mediated induction of target genes is known to be associated with human cancers (Hayes and McMahon, 2009) and its regulation by p62 is responsible for development of human hepatocellular carcinoma (Inami et al., 2011; Takamura et al., 2011). Advances in phenotypic screening provide a novel opportunity to identify additional mechanisms of resistance to autophagy inhibition. Investments in this area of research will allow us to better predict how cancers may circumvent defective autophagy even when combined with current therapies.

## Hurdles to Successful Pharmacological Inhibition of Autophagy

To date, lysomotropic agents such as CQ and hydroxychloroquine (HCQ) are the only candidates undergoing clinical assessment for inhibition of autophagy in cancer. CQ/HCQ have been used in malaria and rheumatologic disorders; they have been repurposed in combination with other agents for treatment of cancers. In numerous studies, CQ in combination with other agents showed beneficial outcomes (Levy et al., 2017; Amaravadi et al., 2019). Additionally, more potent lysosomal inhibitors have been developed, such as Lys05, DQ661, DC661, and ROC-325 (McAfee et al., 2012; Rebecca et al., 2017; Nawrocki et al., 2019; Rebecca et al., 2019). Although some studies with CQ/HCQ suggest clinical benefits, their potency and specificity toward autophagy pathway inhibition remain outstanding concerns (Maycotte et al., 2012). The quest to identify more specific autophagy modulators has driven efforts targeting earlier steps of the pathway (depicted in Figure 1). Most prominent are inhibitors of the Class III lipid kinase Vps34 (patent WO2018144791; Dowdle et al., 2014; Ronan et al., 2014; Bago et al., 2015; Honda et al., 2016; Noman et al., 2020) and the upstream kinases ULK1/2 (Egan et al., 2015; Petherick et al., 2015; Martin K. R. et al., 2018; Chaikuad et al., 2019). SB02024, developed by Sprint Bioscience together with SAR-405 by Sanofi Pharma revealed that inhibition of Vps34 attenuated tumor growth and extended survival in multiple pre-clinical models (Noman et al., 2020). Consistent with genetic observations, pharmacological Vps34 inhibition promoted tumor infiltration by NK and CD8^+^ T cells. It also demonstrated synergy with immune checkpoint inhibitors such as anti-PD-L1 or PD-1. Further down the pathway, inhibitors of the E3-like enzyme ATG7 and the ATG4 family of cysteine proteases present intriguing opportunities (Akin et al., 2014; Kurdi et al., 2017; Liu et al., 2018; Fu et al., 2019; Huang et al., 2020). Nonetheless, genetic models still comprise the large majority of evidence supporting more specific nodes of autophagy as targets. Beyond canonical members of the pathway, genetic phenotypic screens using engineered reporter cell lines have provided new insights for modulation of autophagic flux (DeJesus et al., 2016; Morita et al., 2018; Kerins et al., 2019; Shoemaker et al., 2019). In addition, pharmacological screens with the same cellular tools have revealed inhibitors with new mechanisms of action. For instance, autogramins were identified by an image-based phenotypic screen in EGFP-LC3 overexpressing MCF7 cells (Laraia et al., 2019). Autogramins selectively target GRAMD1A, which is required for autophagosome biogenesis by modulating cholesterol distribution around autophagosome initiation site. For all the above examples (including canonical autophagy genes), a number of unknowns still exist and require improved understanding in order to make meaningful progress for pharmacological modulation of autophagy. Below, we discuss some of these knowledge gaps.

### Identifying Accurate Pharmacodynamic and Predictive Biomarkers of Autophagic Flux

As detailed above, inhibiting autophagy has revealed multiple molecular outcomes which cumulatively impact tumor progression. However, there is a paucity of accurate biomarkers to quantify therapeutic perturbation of autophagy. Classically, accumulation of autophagy receptors such as p62 is acknowledged as a direct pathway biomarker. More recently, immunomodulatory proteins such as MHC-I, TRIF, RIPK1, RIPK3, and STING have shown to be directly modulated by - and in turn impact - autophagy (Matsuzawa-Ishimoto et al., 2017; Samie et al., 2018; Gui et al., 2019; Lim et al., 2019; Liu et al., 2019; Yamamoto et al., 2020). Measuring turnover of autophagic cargo is a valuable cell-associated readout; however, we now appreciate that different cell types within a complex microenvironment exhibit varying kinetics and dynamics of autophagic flux. Additionally, the need for multiple tissue biopsies to measure cargo turnover over course of a treatment poses practical challenges in a therapeutic setting. Thus, identification of peripheral or biofluid-based surrogates would be highly valuable in understanding the magnitude and durability of autophagy inhibition generated by a therapeutic agent. For example, CCL5/RANTES was discussed as a peripheral biomarker for Vps34 inhibition (Mgrditchian et al., 2017; Noman et al., 2020). Although CCL5 is proposed as a direct target of autophagy, it is also a component of the interferon response that is consistently shown to be enhanced upon suppression of autophagy, thus complicating its interpretation as a bona fide autophagic substrate (Martin P. K. et al., 2018; Samie et al., 2018; Wang Y. T. et al., 2020). Additionally, metabolic profiling in pre-clinical models suggest non-essential amino acids Arginine and Alanine as viable circulating biomarkers (Sousa et al., 2016; Poillet-Perez et al., 2018). It will be valuable to determine whether these candidates are 1) meaningful PD biomarkers in a clinical setting, and 2) reflect the kinetics of pharmacological autophagy inhibition. Since the above studies generate chronic or genetic loss of autophagy, a return of autophagic flux is not measured. It is more likely that intermittent modulation of autophagy will be utilized via pulsatile dosing in patients, as systemic, long-term suppression of this pathway may not be desirable. Identification of facile biomarkers that faithfully report inhibition as well as normalization of autophagic flux will prove highly valuable in evaluating therapeutic options.

Accumulating evidence supports autophagy as a mechanism for resistance against targeted, radiation and chemotherapies (Santana-Codina et al., 2017). More recent demonstration of autophagy-mediated resistance to MAP-kinase pathway inhibition in pancreatic cancer have prompted clinical assessment of chloroquine in combination with trametinib (Bryant et al., 2019; Kinsey et al., 2019) [Clinicaltrials.gov NCT03979651]. Emerging pre-clinical studies demonstrate a critical role for autophagy pathway genes in immunosuppression and immune-evasion by cancer. Intriguingly, this is driven by autophagy in the tumor cell as well as components of the TME including myeloid cells and T lymphocytes (Wei et al., 2016; Cunha et al., 2018; DeVorkin et al., 2019; Yamamoto et al., 2020). To identify patients who would benefit most from autophagy inhibition, it will be critical to identify determinants of elevated autophagic flux and measure their correlation with treatment-associated disease progression.

### Non-Canonical Roles of Autophagy Pathway Genes

A growing number of genes associated with autophagic flux also perform autophagy-independent functions. For instance, the Class III PI-3 Kinase Vps34 is well known to regulate endocytic sorting as well as autophagosome formation (Rostislavleva et al., 2015; Stjepanovic et al., 2017). The initiation complex kinases ULK1 and 2 are involved in autophagy-independent lysosomal targeting of ferritin, stress granule degradation, ER-Golgi trafficking of cargo and axon guidance (Joo et al., 2016; Goodwin et al., 2017; Wang et al., 2018; Wang et al., 2019). In phagocytes, components of the Vps34 complex III, autophagosome elongation and maturation machineries have also shown to drive LAP and endocytosis (LANDO) (Cunha et al., 2018; Heckmann et al., 2019). ATG5, ATG16L1, and ATG4 have been implicated in secretory pathways such as membrane exocytosis, leaderless cytokine secretion and exosome release (Murrow et al., 2015; Zhang et al., 2015; Guo et al., 2017; Keller et al., 2020). These non-canonical roles may well be consequential for immunomodulation by autophagy-related genes in the TME.

### Comparing Genetic Models With Pharmacological Inhibition

Genetic mouse models clearly demonstrate a role for autophagy genes in mammalian development as well as tumor progression (reviewed in Kuma et al., 2017; Table 1). Broad functions of Vps34 and ULK1/2 are consistent with embryonic lethality conferred by their germline deficiency. In contrast, genes involved with the autophagosome elongation machinery consistently exhibit perinatal lethality. Thus, even though enhanced autophagy is associated with therapeutic resistance, immunosuppression and disease progression in cancer, the margin of safety should be a key consideration when assessing pharmacological inhibition of autophagy. As highlighted by the murine models discussed above, limitations of genetic loss-of-function prevent a complete assessment of therapeutic autophagy modulation. These include the inability to tune pathway inhibition, the constitutive deletion of targeted gene(s) and the inability to rescue or re-introduce autophagy following its inhibition. These may be particularly important features consider, since it is expected that complete, chronic inhibition of the pathway will likely be detrimental to a durable immune response against cancer. Indeed, recent evidence suggests that pharmacological inhibition of autophagy does not compromise adaptive immunity, consistent with the normal development of mice harboring hypomorphic loss-of-function in core genes such as *Atg16L1* (Cadwell et al., 2008; Hubbard-Lucey et al., 2014; Starobinets et al., 2016; Noman et al., 2020). Moreover, the developmental roles of autophagy-related genes suggest that genetic loss-of-function models do not recapitulate the phenotypic outcomes of pharmacological inhibition, which is transient and incomplete. Thus, careful phenotypic assessment of cellular phenotypes upon pharmacological versus genetic inhibition of autophagy is necessary to delineate which outcomes are consequences of disrupted cell development vs. effector function.

## Concluding Remarks

Novel insights into the immunomodulatory functions of autophagy have driven a resurgence of interest in its pharmacological modulation for numerous diseases. While augmenting autophagy is a relevant therapeutic avenue for autoimmunity, neuroinflammation and chronic inflammatory diseases, its role in cancer has remained pleiotropic. In the setting of established disease, sustained autophagy is acknowledged as a critical mechanism for treatment resistance and immune-evasion. Successful modulation of autophagy will depend on pharmacological approaches which safely diminish autophagic flux to promote meaningful immune responses against cancer, while at the same time allowing for the emergence of durable protection as determined by antigen-specific cellular immunity. The growing number of pharmacological and genetic approaches to modulate autophagy predicts a promising future for its therapeutic targeting to benefit patients battling cancer.

## Author Contributions

All authors directly contributed to the content of the work and approved its publication.

## Conflict of Interest

JL and AM are employees of Genentech, Inc. and shareholders in Roche.
